# Genetic Background Behind the Amino Acid Profiles of Fermented Soybeans Produced by Four *Bacillus* spp.

**DOI:** 10.4014/jmb.2012.12051

**Published:** 2021-01-22

**Authors:** Mihyun Jang, Do-Won Jeong, Ganghun Heo, Haram Kong, Cheong-Tae Kim, Jong-Hoon Lee

**Affiliations:** 1 Department of Food Science and Biotechnology, Kyonggi University, Suwon 16227, Republic of Korea; 2 Department of Food and Nutrition, Dongduk Women’s University, Seoul 02748, Republic of Korea; 3 Nongshim Co., Ltd., Seoul 07065, Republic of Korea

**Keywords:** Soybean fermentation, *Bacillus*, γ-aminobutyric acid, *gadA/B*, *rocE*, *puuD*

## Abstract

Strains of four *Bacillus* spp. were respectively inoculated into sterilized soybeans and the free amino acid profiles of the resulting cultures were analyzed to discern their metabolic traits. After 30 days of culture, *B. licheniformis* showed the highest production of serine, threonine, and glutamic acid; *B. subtilis* exhibited the highest production of alanine, asparagine, glycine, leucine, proline, tryptophan, and lysine. *B. velezensis* increased the γ-aminobutyric acid (GABA) concentration to &gt;200% of that in the control samples. *B. sonorensis* produced a somewhat similar amino acid profile with *B. licheniformis*. Comparative genomic analysis of the four *Bacillus* strains and the genetic profiles of the produced free amino acids revealed that genes involved in glutamate and arginine metabolism were not common to the four strains. The genes *gadA/B* (encoding a glutamate decarboxylase), *rocE* (amino acid permease), and *puuD* (γ-glutamyl-γ-aminobutyrate hydrolase) determined GABA production, and their presence was species-specific. Taken together, *B. licheniformis* and *B. velezensis* were respectively shown to have high potential to increase concentrations of glutamic acid and GABA, while *B. subtilis* has the ability to increase essential amino acid concentrations in fermented soybean foods.

## Introduction

Several types of fermented soybean food are consumed in Korea and representative examples are ganjang (soy sauce) and doenjang (soybean paste). The traditional production of ganjang and doenjang starts from the manufacture of meju. Meju is made by soaking, steaming, crushing, and molding soybeans into blocks, followed by spontaneous fermentation for 2–3 months. The ripened meju is mixed with brine and ripened for a further 2–3 months, then the liquid portion of the mixture is separated, resulting in a traditional type of ganjang. The remaining solid portion is subsequently mashed and fermented for >6 months and becomes quality doenjang [1]. Ripened meju is used as a starter culture as well as a nutrient and flavor source for fermented food production in Korea [2].

Understanding of traditional fermented soybean foods has long been a research theme of food scientists in Korea. Several studies including microbial community analysis have provided basic insight for accelerated ripening, quality assurance, and flavor enhancement of fermented soybean foods. Early microbial studies isolated and identified microorganisms exhibiting strong amylase, protease, and lipase activities that can contribute to degradation of soybean macromolecules [3-6]. More recent culture-independent microbial community analysis techniques have revealed the presence of a wider variety of microorganisms in the food matrices [7-15]. However, regardless of the analytical methods used, most microbial studies reported that the bacterial genus *Bacillus* and the fungal genus *Aspergillus* were the most populous microorganisms.

Recently, molecular biology techniques and sequence databases have contributed to identification, characterization, and typing of bacteria and increased the number of novel bacterial species [16]. Rapid advances in whole-genome sequencing technologies and analysis pipelines have further enhanced the resolution power of bacterial species and influenced the taxonomic status of closely related identities. This advanced bacterial identification methodology has affected the consolidation and assignment of new taxa in the genus *Bacillus* [17-19]. In this context, we isolated and identified *Bacillus* spp. from 12 meju samples collected from five regions of Korea to determine the predominant *Bacillus* species in meju based on current bacterial taxonomic standards [20]. One hundred and fifty-one *Bacillus* isolates were identified, in the following order of predominance: *B. velezensis*, *B. sonorensis*, *B. subtilis*, and *B. licheniformis*.

The safety-approved *Aspergillus oryzae* is normally used as a starter in the production of commercial fermented soybean foods in Korea. Several studies have employed *Bacillus* spp. in the soybean food manufacturing process, and the selected starters reportedly accomplished their target purposes [21-26], but *Bacillus* spp. have not yet been introduced in mass production. A lack of detailed understanding of the contribution of each *Bacillus* species to soybean food fermentation hinders the introduction of *Bacillus* spp. as starter cultures.

In the current study, we respectively inoculated one strain each of *B. velezensis*, *B. sonorensis*, *B. subtilis*, and *B. licheniformis* into sterilized soybeans and analyzed the free amino acid profiles in the soybean culture samples during fermentation to discern their metabolic traits and provide information for selecting *Bacillus* starter culture candidates for soybean food fermentation. We also performed a comparative genomic analysis of the four *Bacillus* strains to shed light on the genetic profiles of the free amino acids they produce.

## Materials and Methods

### *Bacillus* Strains and Cultures

All selected *Bacillus* strains were isolated from fermented soybean foods or soybeans and their complete genome sequences have been published (Table 1). *B. licheniformis* 14ADL4 (KCTC 33983) was isolated in our laboratory and deposited in the Korean Collection for Type Cultures [27]. *B. sonorensis* SRCM101395 was kindly provided by the Microbial Institute for Fermentation Industry (http://mifi.kr). *B. subtilis* ATCC 21228 (KCCM 40443) was purchased from the Korean Culture Center of Microorganisms (http://kccm.or.kr/). *B. velezensis* JJ-D34 was kindly provided by Prof. Che Ok Jeon, Chung-Ang University, South Korea [28]. All *Bacillus* strains were cultured on Difco tryptic soy agar (BD Diagnostic Systems, USA) and in Difco tryptic soy broth (TSB; BD Diagnostic Systems) at 37°C for 24 h.

### Preparation of *Bacillus* Strain-Inoculated Soybean Samples

Korean soybeans, known as baektae (*Glycine max* L. Merrill), were washed, soaked in equal amounts of water for 18 h at room temperature, and then crushed using a mortar. Fifty grams of the crushed soybeans was placed in 500-ml reagent bottles then autoclaved for 30 min at 121°C. Respective logarithmic-phase *Bacillus* cells cultured in TSB were inoculated into the crushed and sterilized soybeans at 5 × 10^5^ colony-forming units (CFU)/g then mixed thoroughly. Samples were prepared in duplicate and incubated aseptically at 25°C for 30 days along with crushed and sterilized soybean samples as controls. This culture temperature was set to align with future production of fermented soybean foods at room temperature. Samples were collected at days 1, 15, and 30 and stored at −70°C for subsequent chemical and microbiological analyses.

### Viable Cell Count and pH Analysis of Soybean Culture Samples

Five grams of each sample was homogenized with 20 ml sterilized peptone water and filtered through sterilized cheesecloth. The filtrates were spread onto Difco plate count agar (BD Diagnostic Systems) after serial dilution using saline then incubated at 37°C for 24 h to determine viable cell numbers. The pH of the filtrates was measured using a pH meter. All experiments were conducted on three independent samples prepared in the same way.

### Analysis of Free Amino Acids in Soybean Culture Samples

Samples (5 g) were mixed with 50 ml of 75% aqueous EtOH (v/v). The mixture was sonicated for 1 h, soaked at room temperature for 24 h, and then filtered through a 0.2-μm membrane filter (Phenomenex, USA). The filtrate was analyzed by a custom service provided by the National Instrumentation Center for Environmental Management in Korea (http://nicem.snu.ac.kr/). The analyses were performed using the Dionex Ultimate 3000 HPLC system (Thermo Scientific, USA). Chromatographic separation was achieved with a VDSpher 100 C18-E column (150 × 4.6 mm, 3.5 μm; VDS Optilab, Germany). Gradient elution was carried out with sodium phosphate buffer (solvent A; pH 7) and water/acetonitrile/methanol (solvent B; 10:45:45, v/v/v). The following binary mobile phase linear gradients were used: 100% A at 0 min, 95% A at 24 min, 45% A at 25 min, 20% A at 34.5 min, and 95%A at 35 min. The column temperature and flow rate were 40°C and 1 ml/min, respectively. The detection was performed using a fluorescent detector. Two derivatizing agents, OPA (ο-phthaldialdehyde; Agilent, USA) and FMOC (9-fluorenylmethoxycarbonyl chloride; Agilent), were simultaneously used according to the manufacturer’s instructions. Excitation/emission wavelengths were respectively 340/450 nm for OPA-derivatized amino acids and 266/305 nm for FMOC-derivatized amino acids. The concentrations of individual free amino acids were determined using five-point calibration curves of Amino Acid Standard (WAT088122, Waters Corporation, USA). The free amino acid content of two samples prepared in the same conditions was analyzed twice.

### Comparative Genomic Analysis and Metabolic Pathway Prediction of *Bacillus* Strains

Genomic information for comparative genomic analyses of *Bacillus* strains including *B. licheniformis* 14ADL4, *B. sonorensis* SRCM 101395, *B. subtilis* ATCC 21228, and *B. velezensis* JJ-D34 was downloaded from the NCBI database (http://ncbi.nlm.nih.gov/genomes), and the EzBioCloud database (https://www.ezbiocloud.net/). Core-genome and pan-genome analyses were performed using the Efficient Database framework for comparative Genome Analyses using BLASTP score Ratios (EDGAR) [29]. Two genes were considered orthologous when a bidirectional best BLAST hit with a single score ratio value threshold of at least 32% was obtained for orthology estimation. The *B. licheniformis* 14ADL4 genome was used as the reference genome for Venn diagram construction for four-genome analysis using EDGAR. Rapid Annotation using Subsystem Technology (RAST)[30] and the Interactive Pathways Explorer v3 (https://pathways.embl.de/) were used to determine gene contents based on functional subsystem classifications and to estimate the amino acid metabolic pathways. Comparative analyses at the protein level were performed by an all-against-all comparison of the annotated genomes.

### Statistical Analysis

One-way analysis of variance followed by Duncan’s multiple range test was used to evaluate significant differences between the average values obtained in the free amino acid analyses. *p*-value < 0.05 was considered statistically significant. To visualize the differences between the amino acids produced from the sterilized soybeans by the inoculated bacteria, principal component analysis (PCA) was applied with maximum variation rotation. All statistical analyses were performed using the SPSS software package (version 22.0; SPSS, IBM, USA).

## Results

### Growth of Four *Bacillus* Strains in Soybean Cultures and Their Contribution to pH Changes

**Fig. 1:**
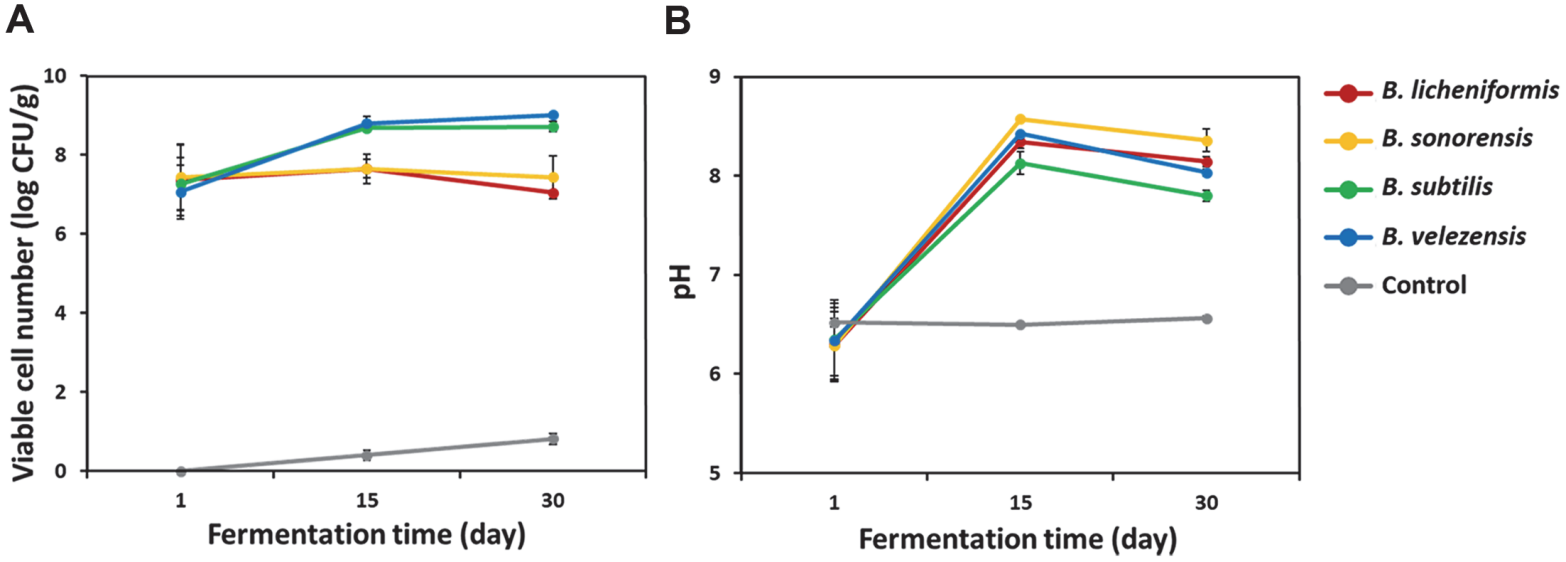
Growth (A) and pH changes (B) of soybean cultures inoculated with four *Bacillus* strains over 30 days of incubation.

Fewer than 10 CFU/g were detected in the control soybean samples at day 30 (Fig. 1A). Most microorganisms present in the soybeans were eliminated by autoclaving (30 min at 121°C). The almost constant pH during the 30-day incubation of control soybean samples demonstrated that few changes occurred in these cultures (Fig. 1B).

In inoculated cultures, there was no distinguishable difference in the cell numbers of the *Bacillus* strains on day 1 (average cell number 2.00 × 10^7^ CFU/g). Until day 15, *B. subtilis* and *B. velezensis* maintained consistent growth, but *B. licheniformis* and *B. sonorensis* did not. At day 30, the number of *B. velezensis* cells (1.02 × 10^9^ CFU/g) was almost 100 times that of *B. licheniformis* (1.10×10^7^ CFU/g); those were the highest and lowest cell numbers in the soybean cultures. The growth rates of the *Bacillus* strains were not sufficient to assert that the highest growth on soybeans among the four *Bacillus* spp. was by *B. velezensis*. According to Jang et al. [20], *B. velezensis* was the most populous *Bacillus* species identified in meju, which provides evidence that *B. velezensis* grows better on soybeans than *B. licheniformis*.

As the fermentation progressed, the samples inoculated with *Bacillus* showed a tendency of pH increase and then decrease. The increase of pH until day 15 can be attributed to the amino acids and amines produced by the degradation of soybean protein, and the pH decrease after day 15 might be caused by acid production by the strains [31].

### Free Amino Acid Production in Soybean Cultures by Four *Bacillus* Strains

Nineteen free amino acids including γ-aminobutyric acid (GABA), a non-proteinogenic amino acid, were quantified in the controls and *Bacillus* strain-inoculated soybean cultures (Table 2). The concentration of free amino acids in the control samples did not change significantly during the 30-day incubation. Sixteen of the 18 identified proteinogenic amino acids were increased in concentration by the growth of the four *Bacillus* spp. A decrease in arginine concentration was detected in all of the inoculated soybean cultures, and a decrease of asparagine concentration was identified in *B. sonorensis* cultures.

After 30 days of culture, *B. licheniformis* showed the highest production of three amino acids (serine, threonine, and glutamic acid), and *B. subtilis* exhibited the highest production of seven amino acids (alanine, asparagine, glycine, leucine, proline, tryptophan, and lysine). Regardless of the inoculated *Bacillus* spp., the amount of glutamic acid produced from the soybean cultures was highest among the 19 amino acids. *B. licheniformis* was the highest producer, with a 47.7-fold increase in glutamic acid concentration at day 30 compared with day 1. The concentration of GABA in the soybean cultures was not significantly changed by *B. subtilis*. However, *B. licheniformis* and *B. sonorensis* decreased the GABA concentration in their soybean cultures to <20% of that in the control samples after 15 days, while *B. velezensis* increased the GABA concentration to >200% of that in the control samples in the same period. No correlation was found between the growth and the amino acid profiles produced by the four *Bacillus* spp. Free amino acid production by the four *Bacillus* spp. might depend on their metabolic traits rather than their biological activities.

### PCA for Free Amino Acids Produced by the Four *Bacillus* Strains

**Fig. 2:**
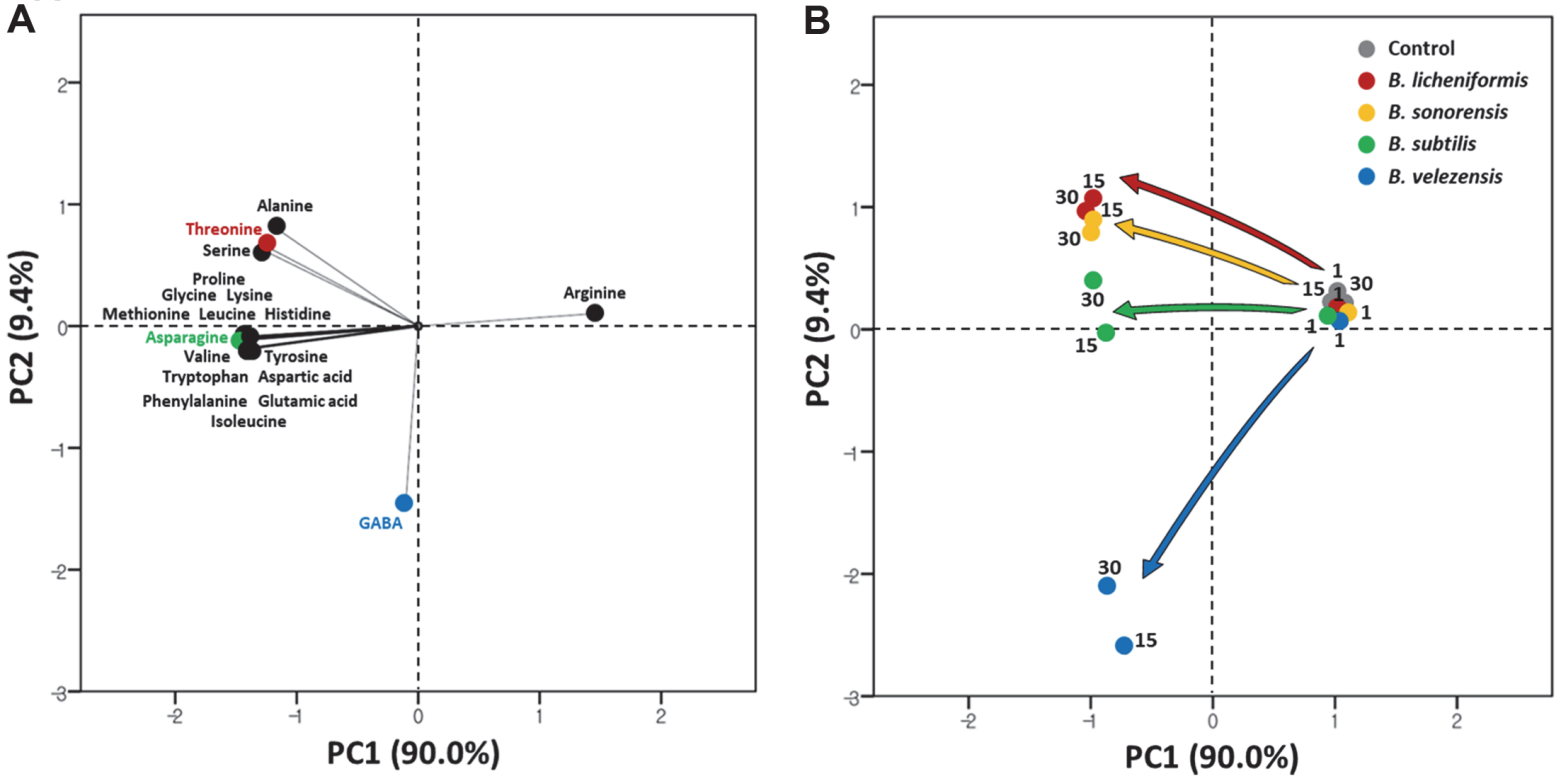
Principal component analysis loadings from four *Bacillus* spp.-inoculated soybean samples over 30 days of incubation for (A) amino acid concentrations and (B) factor scores. Numbers indicate the incubation time of samples in days.

Statistics on the 19 free amino acids produced from sterilized soybeans by the growth of four *Bacillus* spp. were subjected to PCA (Fig. 2). Arginine concentration decreased in the cultures of the four *Bacillus* spp. and this amino acid is located in the positive part of the PC1 dimension in the PCA factor loading plot, while the other 18 free amino acids were negatively correlated with PC1 (Fig. 2A). GABA increased only in the cultures of *B. velezensis* and is isolated in the PCA factor loading plot.

The PCA scores of the four *Bacillus* spp.-inoculated soybean cultures after 1, 15, and 30 days of incubation are shown in Fig. 2B. All factor scores at day 1 clustered together with those of the control samples, indicating that the amino acid production by *Bacillus* spp. in a short-term soybean culture was not sufficient to indicate the specific characteristics of the strains. The factor scores of *Bacillus* spp.-inoculated samples developed in different directions during incubation, but the factor scores on day 30 compared with day 15 do not exhibit the same dramatic differences as those on day 15 compared with day 1. Fifteen-day soybean cultures of the four *Bacillus* spp. may be sufficient to discern their specific characteristics in amino acid production.

The factor scores of *B. licheniformis*-inoculated samples developed in a similar direction as those of the *B. sonorensis*-inoculated samples. The production of alanine, serine, and threonine determined the direction of *B. licheniformis*- and *B. sonorensis*-inoculated samples in the factor score plot. *B. licheniformis* exhibited the highest production of serine and threonine and *B. sonorensis* was the next highest producer of both amino acids. The increase ratios of alanine by *B. licheniformis* and *B. sonorensis* were between those of *B. subtilis* and *B. velezensis*. The *B. licheniformis* and *B. sonorensis* strains produced somewhat similar amino acid profiles in the soybean cultures. The production of 14 amino acids including the essential amino acids isoleucine, leucine, methionine, phenylalanine, tryptophan, valine, and lysine determined the direction of *B. subtilis*-inoculated samples in the factor score plot. The amount of asparagine produced by *B. subtilis* during the 30-day-incubation was >2.5 times greater than that of the other strains. Asparagine may be the crucial amino acid determining the direction of the factor scores of *B. subtilis*. The production of GABA determined the locations of *B. velezensis* factor scores, which are distant from those of the other species. The profile differences of the amino acids produced by the four *Bacillus* strains instigated further studies to illuminate the determinants and characteristics involved in amino acid production dependent on species.

### Genomic Insight into the Amino Acid Profiles of Soybean Cultures Produced by Four *Bacillus* Strains

**Fig. 3:**
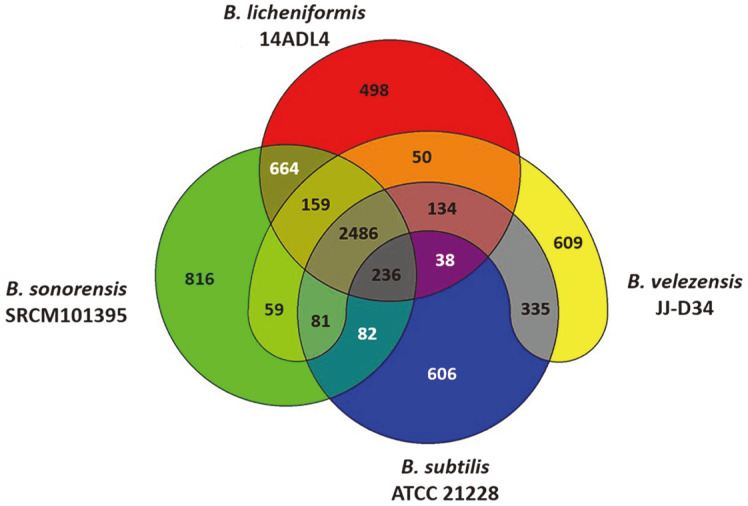
Venn diagram showing the pan-genome of four *Bacillus* spp. strains. Overlapping regions represent common coding sequences (CDSs) shared between the genomes. The numbers outside the overlapping regions indicate the numbers of CDSs in each genome without homologs in the other genomes.

The gene pools shared by the genomes of the four *Bacillus* strains are depicted in a Venn diagram (Fig. 3). These four strains share 2,486 protein-coding sequences (CDSs) in their core genome, corresponding to 53.9%–63.6% of their CDSs. The genome of *B. velezensis* JJ-D34 has 8.9% unique CDSs (*i.e.*, ones that are absent from the other three *Bacillus* genomes). The proportions of unique CDSs in the genomes of *B. licheniformis* 14ADL4, *B. sonorensis* SRCM101395, and *B. subtilis* ATCC 21228 are 7.3%, 11.9%, and 8.8%, respectively.

**Fig. 4:**
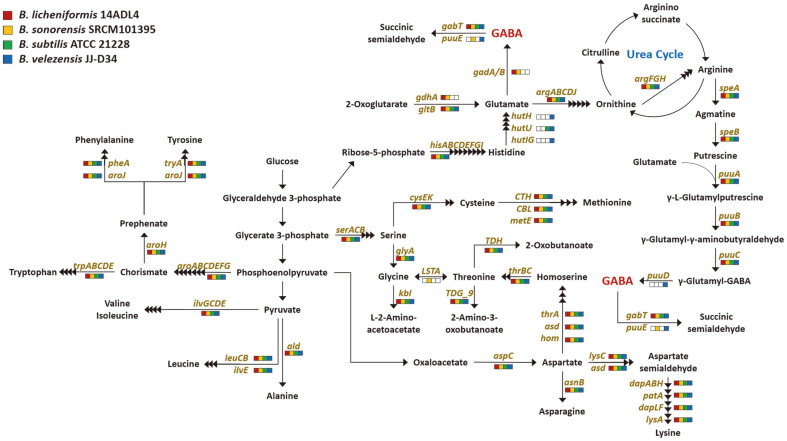
Identification of genes involved in the amino acid metabolism of four *Bacillus* spp. strains.

Most of the amino acid metabolic pathway genes were identified in all four genomes (Fig. 4). However, genes involved in glutamate and arginine metabolism were not shared by all four strains. The *B. licheniformis* and *B. sonorensis* strain genomes contain homologs for the conversion of 2-oxoglutarate to GABA via glutamate, including a *gadA/B* (glutamate decarboxylase) homolog encoding an enzyme that can convert glutamate to GABA [32]. Thus, the *B. licheniformis* and *B. sonorensis* strains may be able to produce GABA via this pathway. All four strains possess a *gabT* (GABA aminotransferase) homolog; this enzyme has been reported to decompose GABA into succinic semialdehyde [33]. The decrease of GABA in the soybean cultures of *B. licheniformis* and *B. sonorensis* can be attributed to their possession of *gabT*, but the metabolism in *B. subtilis* and *B. velezensis* is not clearly explained.

**Fig. 5:**
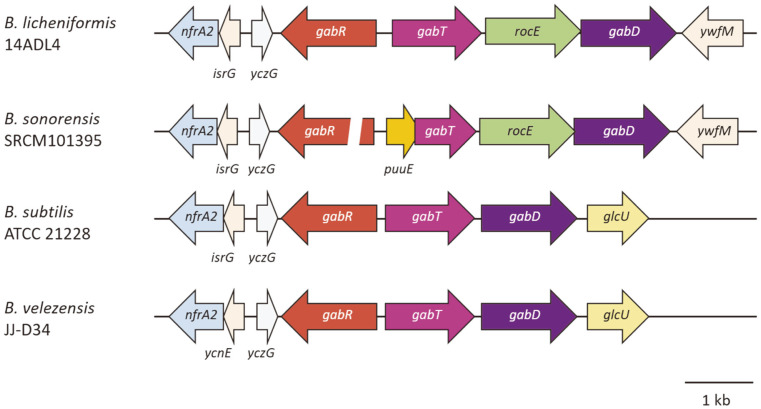
Structures of genes surrounding the annotated *gabT* gene in four *Bacillus* genomes. Abbreviations: *gabR*, HTH-type transcriptional regulatory protein-encoding gene; *gabT*, GABA aminotransferase-encoding gene; *rocE*, amino acid permease-encoding gene; *gabD*, succinate-semialdehyde dehydrogenase-encoding gene.

*B. velezensis* JJ-D34 has homologs for the conversion of histidine to glutamate, but the amount of glutamate identified in its soybean culture was the lowest among the soybean cultures of the four *Bacillus* spp. All four strains possess homologs of genes to produce γ-glutamyl-γ-aminobutyric acid (γ-glutamyl-GABA) via putrescine from arginine, while only *B. velezensis* has a *puuD* (γ-glutamyl-γ-aminobutyrate hydrolase) homolog that may convert γ-glutamyl-GABA to GABA [34]. The *puuD* homolog of *B. velezensis* strain JJ-D34 was annotated as a glutamine amidotransferase-encoding gene in the NCBI database (GenBank Accession No. AKF29542.1). A *puuE* (4-aminobutyrate-2-oxoglutarate transaminase) homolog, encoding an enzyme known to convert GABA to succinic semialdehyde, was identified in the *B. sonorensis* and *B. velezensis* genomes [35]. The highest amount of GABA was identified in the *B. velezensis* cultures, indicating that the pathway for conversion of arginine to GABA was active, while the pathway for conversion of GABA to succinic semialdehyde was inactive. In the case of *B. sonorensis* SRCM101395, the annotated *puuE* is located between *gabR* (HTH-type transcriptional regulatory protein) and a *gabT* homolog and is smaller than *puuE* from *B. velezensis* JJ-D34 (513 bp compared with 1,269 bp) (Fig. 5). Additionally, deletions were identified in the *gabR* and *gabT* homologs of *B. sonorensis* SRCM101395 compared with the corresponding genes from the other strains. Further studies are required to show whether the *puuE* homologs of *B. sonorensis* and *B. velezensis* contribute to the conversion of GABA.

We could not find any clues as to whether the *gabT* homologs of *B. subtilis* and *B. velezensis* are involved in the conversion of GABA to succinic semialdehyde. We analyzed the flanking regions of the *gabT* homologs in the genomes of the four strains used in this study and found *rocE* (amino acid permease) homologs located downstream of the *gabT* homologs in the genomes of the *B. licheniformis* and *B. sonorensis* strains (Fig. 5). The *gabT* and *rocE* homologs form a putative operon with *gabD* (succinate-semialdehyde dehydrogenase). The expression of a GABA transporter gene might contribute to the decrease of GABA in the soybean cultures of *B. licheniformis* and *B. sonorensis*. *B. subtilis* ATCC 21228 possesses homologs of neither *gadA/B* nor *puuD*. The highest GABA production among the four *Bacillus* strains, by *B. velezensis* JJ-D34, may be attributed to its possession of *puuD*.

To determine whether the GABA-producing characteristics of the four *Bacillus* strains studied in this work can be extended to species-specific characteristics, we checked for the presence of five gene homologs supposedly involved in GABA metabolism in the complete genomes of many strains of the four *Bacillus* spp. (Table 3). Among 135 *B. subtilis* strains, only strain BEST7613 (AP012495.1) possesses an annotated gad, but its product has only 12.6% amino acid sequence homology with *B. licheniformis* DSM 13^T^ glutamate decarboxylase (data not shown). Thus, the annotated *gad* of strain BEST7613 may not endow glutamate decarboxylase function. With few exceptions, we confirmed the species-specific presence of *gadA/B*, *rocE*, *puuD*, and *puuE* in the four *Bacillus* spp. Among the 135 *B. subtilis* strains, six possess *puuE* homologs and their flanking regions exhibit similar gene structures to that in *B. velezensis* (data not shown). The presence of *puuE* homologs in the six *B. subtilis* strains might be the result of genetic recombination between *B. subtilis* and *Bacillus* strains possessing *puuE* homologs. The presence of *gadA/B*, *rocE*, and *puuD* can be used as a biomarker for the selection of GABA-producing starter culture candidates.

## Discussion

Fermented foods are consumed all over the world. The application of starter cultures has been reported to provide technological, nutritional, and health advantages in terms of the final product composition. The first stage in designing a starter culture for a fermented food is to characterize the microbiota of the food matrix of interest and then select strains that are best suited to that environment. Traditionally, in starter selection, significant emphasis was placed on the technological phenotypes of strains, including growth performance or activity, flavor testing, and matrix formation analysis [36]. Sometimes, the selected strain does not reflect the true structure of the microbiota in the fermentation and fails to display adequate performance in the target fermentation process.

Traditional *Bacillus*-involved fermented soybean foods are consumed in many countries in Asia and Africa as a good source of protein, and *B. subtilis* has been known as the major fermenting species [37]. However, the progress of molecular taxonomy and next-generation sequencing technologies has created the need to shed new light on the major fermenters based on current taxonomic standards and their functional differences in soybean food fermentation, and will serve as a cornerstone for the selection of useful strains with target characteristics.

Soybeans are one of the most popular plant-based proteins used in food products, with a protein content of 35%–40% on a dry weight basis [38]. Therefore, proteolysis of soybean protein is one of the most important processes in terms of primary flavor development in soybean fermentation. Free amino acids have been reported to contribute directly to taste perception, and act as a precursor of flavor enhancement [39].

Several studies on the impact of fermentation on soybean protein have been performed and concluded that the profile of free amino acids in fermented products not only depends on the starter culture used, but also on the soybean variety used for fermentation [40]. In this study, we found differences in amino acid profiles of soybean cultures produced by strains of four *Bacillus* spp. and clues to the mechanisms of species-specific production through comparative genomic analysis. Our results will contribute to proper selection of *Bacillus* starter culture candidates for soybean food fermentation in accordance with the goals of each producer. *B. licheniformis* can be used to enhance the concentration of glutamic acid, which is a key compound that determines umami taste of fermented soybean foods including soy sauce [41]. *B. subtilis* has good potential to increase the concentrations of the essential amino acids isoleucine, leucine, methionine, phenylalanine, tryptophan, valine, and lysine in fermented soybean foods. *B. velezensis* was shown to produce large amounts of GABA, a well-known bioactive compound that increases the value of fermented soybean products. Further metabolic studies with multiple strains of the four *Bacillus* spp. are required to prove that the results of this study can be extended to the characteristics of each species.

## Figures and Tables

**Table 1:** Genomic features of *Bacillus* strains used in this study.

Strain	*B. licheniformis* 14ADL4	*B. sonorensis* SRCM101395	*B. subtilis* ATCC 21228	*B. velezensis* JJ-D34
Chromosome size (bp)	4,332,232	4,832,293	4,141,030	4,105,955
Chromosome G+C content (%)	45.9	45.2	43.5	46.2
No. of plasmids	–	–	1	–
Plasmid size (bp)	–	–	85,618	–
Plasmid G+C content (%)	–	–	38.1	–
Predicted CDSs	4,273	4,609	4,151	3,907
No. of rRNAs	24	24	30	27
No. of tRNAs	81	85	87	86
Contigs	1	1	2	1
Origin	Doenjang	Food	Soybeans	Doenjang
Country	Korea	Korea	USA	Korea
Accession no.	NZ_CP026673.1	NZ_CP021920.1	NZ_CP020023.1, NZ_CP020024.1	NZ_CP011346.1
Reference	[27]	–	–	[28]

Genomic features were referred from analysis results in the NCBI database.

**Table 2:** Free amino acid profiles in soybean cultures of four *Bacillus* spp. strains at days 1, 15, and 30. (unit: mg/kg)

Amino acid	Control				*B. licheniformis* 14ADL4				*B. sonorensis* SRCM101395				*B. subtilis* ATCC 21228				*B. velezensis* JJ-D34		
								
	Day 1	Day 15	Day 30		Day 1	Day 15	Day 30		Day 1	Day 15	Day 30		Day 1	Day 15	Day 30		Day 1	Day 15	Day 30
Neutral amino acid																			
Alanine	160.67^a^	169.17^a^	191.52^a^		167.67^a^	2433.26^bc^	3049.71^cd^		165.75^a^	2034.47^b^	2382.13^bc^		158.93^a^	3388.02^d^	3799.28^d^		167.77^a^	752.88^a^	937.89^a^
Asparagine	147.08^abc^	145.28^abc^	147.88^abc^		149.68^abc^	243.65^bcd^	285.11^cd^		141.79^abc^	62.36^ab^	26.05^a^		127.47^abc^	522.64^e^	770.49^f^		143.57^abc^	245.25^bcd^	392.10^de^
Glycine	46.11^a^	50.93^a^	48.09^a^		52.93^a^	1172.65^d^	1519.46^e^		50.77^a^	757.26^bc^	960.31^cd^		53.39^a^	1970.79^f^	2065.46^f^		57.75^a^	494.35^b^	690.44^bc^
Isoleucine	41.21^a^	42.19^a^	50.69^a^		46.79^a^	2767.39^cd^	3318.77^d^		47.77^a^	1913.29^b^	2386.80^bc^		57.67^a^	3155.71^cd^	3562.05^d^		64.04^a^	3113.75^cd^	3284.90^d^
Leucine	55.26^a^	55.82^a^	67.84^a^		74.86^a^	5695.12^cd^	6756.67^de^		73.82^a^	4159.09^b^	4858.47^bc^		85.40^a^	6710.01^de^	7463.43^e^		102.82^a^	5299.35^bc^	5494.63^cd^
Methionine	20.21^a^	19.35^a^	21.99^a^		28.04^a^	1150.18^def^	1191.66^ef^		28.80^a^	860.04^cd^	790.27^bc^		31.21^a^	1286.70^f^	950.86^cde^		32.97^a^	1030.11^cdef^	526.32^b^
Phenylalanine	79.03^a^	81.29^a^	93.30^a^		93.67^a^	4637.11^cd^	5103.84^d^		92.25^a^	3540.35^b^	3731.90^bc^		107.18^a^	5339.07^d^	5745.45^d^		119.31^a^	5349.07^d^	5285.23^d^
Proline	56.90^a^	32.72^a^	33.61^a^		31.88^a^	2346.05^b^	2958.76^bc^		31.20^a^	2947.26^bc^	3079.49^bc^		47.95^a^	4016.13^bc^	4227.06^c^		58.32^a^	3167.93^bc^	3739.25^bc^
Serine	51.44^a^	52.83^a^	61.08^a^		57.62^a^	1524.70^e^	2006.15^f^		57.23^a^	1165.90^d^	1468.59^e^		74.70^a^	753.50^c^	1121.47^d^		77.51^a^	398.49^b^	690.07^c^
Threonine	45.51^a^	49.16^a^	60.11^a^		53.44^a^	2411.26^f^	2434.95^f^		50.72^a^	1140.35^de^	1340.80^e^		71.26^a^	886.79^cd^	1092.44^de^		75.19^a^	434.53^b^	648.53^bc^
Tryptophan	176.27^a^	180.59^a^	190.18^a^		194.04^a^	1224.23^cd^	1325.69^de^		196.15^a^	763.09^b^	832.84^bc^		191.29^a^	1825.04^f^	1732.31^ef^		208.42^a^	1672.99^ef^	1304.24^de^
Tyrosine	63.91^a^	65.23^a^	80.33^a^		73.41^a^	3213.56^cd^	3280.44^cd^		70.59^a^	2439.95^b^	2628.89^bc^		89.61^a^	3681.09^d^	3218.17^cd^		97.86^a^	2993.13^bcd^	2982.32^bcd^
Valine	54.58^a^	56.74^a^	65.65^a^		65.66^a^	3410.67^bcd^	3977.86^cde^		66.21^a^	2526.52^b^	2988.66^bc^		78.99^a^	4482.40^de^	5062.84^e^		89.25^a^	4398.59^de^	4648.09^e^
Acidic amino acid																			
Aspartic acid	53.01^a^	56.12^a^	60.18^a^		60.78^a^	2803.34^d^	3665.16^e^		59.34^a^	1924.10^bc^	2601.13^cd^		78.39^a^	3158.57^de^	3700.52^e^		92.02^a^	1220.74^b^	1703.74^b^
Glutamic acid	404.74^a^	410.35^a^	434.01^a^		420.44^a^	18077.65^e^	20053.26^e^		415.27^a^	10107.50^c^	11267.42^c^		445.43^a^	12783.35^cd^	14715.05^d^		472.16^a^	5457.30^b^	6723.10^b^
Basic amino acid																			
Arginine	1046.67^d^	1059.49^d^	1070.95^d^		1116.34^d^	19.01^a^	17.62^a^		1122.74^d^	32.39^a^	35.29^a^		1039.68^d^	293.31^bc^	278.52^bc^		1139.26^d^	97.20^ab^	371.46^c^
Histidine	53.06^a^	54.18^a^	60.52^a^		71.27^a^	2107.06^d^	1990.60^cd^		57.10^a^	1619.26^bc^	1290.84^b^		63.51a^a^	2136.37^d^	1968.19^cd^		74.30^a^	2065.33^cd^	1599.05^bc^
Lysine	73.71^a^	85.49^a^	95.88^a^		93.60^a^	5057.28^cd^	6007.25^de^		108.06^a^	3823.86^b^	4168.82^bc^		122.86^a^	6921.71^ef^	7272.85^f^		134.86^a^	4945.31^bcd^	4509.57^bc^
Functional amino acid																			
GABA	232.27^b^	257.63^b^	285.51^b^		273.72^b^	40.36^a^	49.14^a^		275.59^b^	53.89^a^	60.93^a^		303.79^b^	270.59^b^	309.81^b^		316.22^b^	668.28^c^	698.54^c^

Different superscripts within a row denote a significant difference between mean values (*p* < 0.05) according to Duncan’s multiple range test.

**Table 3:** Presence of potential genes involved in GABA production (analysis of data available in March 2020).

Species	Number of strains					

	Complete genomes published	*gadA/B* possessor	*gabT* possessor	*rocE* possessor	*puuD* possessor	*puuE* possessor
*B. licheniformis*	27	27	27	27	-	-
*B. sonorensis*	1	1	1	1	-	(1)
*B. velezensis*	110	-	110	-	110	110
*B. subtilis*	135	(1)	135	-	-	6

Numbers in parentheses indicate the number of strains having the annotated genes and the genes may have a different function.

## References

[1] Park KY, Hwang KM, Jung KO, Lee KB (2002). Studies on the standardization of *doenjang* (Korean soybean paste). J. Korean Soc. Food Sci. Nutr..

[2] Shin D, Jeong D (2015). Korean traditional fermented soybean products: Jang. J. Ethnic Foods.

[3] Choi KK, Chi CB, Ham SS, Lee DS (2003). Isolation, identification and growth characteristics of main strain related to meju fermentation. J. Korean Soc. Food Sci. Nutr..

[4] Kang MJ, Kim SH, Joo HK, Lee GS, Yim MH (2000). Isolation and identification of microorganisms producing the soy protein hydrolyzing enzyme from traditional *mejus*. J. Korean Soc. Agric. Chem. Biotechnol..

[5] Kwon OJ, Kim JK, Chung YK (1986). The characteristics of bacteria isolated from ordinary Korean soy sauce and soybean paste. J. Korean Agri. Chem. Soc..

[6] Yoo SK, Cho WH, Kang SM, Lee SH (1999). Isolation and identification of microorganisms in Korean traditional soybean paste and soybean sauce. Korean J. Appl. Microbiol. Biotechnol..

[7] Cho KM, Seo WT (2007). Bacterial diversity in Korean traditional soybean fermented foods (*doenjang* and *ganjang*) by 16S rRNA gene sequence analysis. Food Sci. Biotechnol..

[8] Jung JY, Lee SH, Jeon CO (2014). Microbial community dynamics during fermentation of doenjang-meju, traditional Korean fermented soybean. Int. J. Food Microbiol..

[9] Jung WY, Jung JY, Lee HJ, Jeon CO (2016). Functional characterization of bacterial communities responsible for fermentation of *doenjang*: a traditional Korean fermented soybean paste. Front. Microbiol..

[10] Kim TW, Lee JH, Kim SE, Park MH, Chang HC, Kim HY (2009). Analysis of microbial communities in *doenjang*, a Korean fermented soybean paste, using nested PCR-denaturing gradient gel electrophoresis. Int. J. Food Microbiol..

[11] Kim YS, Jeong DY, Hwang YT, Uhm TB (2011). Bacterial community profiling during the manufacturing process of traditional soybean paste by pyrosequencing method. Korean J. Microbiol..

[12] Kim YS, Kim MC, Kwon SW, Kim SJ, Park IC, Ka JO (2011). Analyses of bacterial communities in meju, a Korean traditional fermented soybean bricks, by cultivation-based and pyrosequencing methods. J. Microbiol..

[13] Lee JH, Kim TW, Lee H, Chang HC, Kim HY (2010). Determination of microbial diversity in *meju*, fermented, cooked soya beans, using nested PCR-denaturing gradient gel electrophoresis. Lett. Appl. Microbiol..

[14] Lee SY, Kim HY, Lee S, Lee JM, Muthaiya MJ, Kim BS (2012). Mass spectrometry-based metabolite profiling and bacterial diversity characterization of Korean traditional *meju* during fermentation. J. Microbiol. Biotechnol..

[15] Nam YD, Lee SY, Lim SI (2012). Microbial community analysis of Korean soybean pastes by next-generation sequencing. Int. J. Food Microbiol..

[16] Franco-Duarte R, Černáková L, Kadam S, Kaushik KS, Salehi B, Bevilacqua A (2019). Advances in chemical and biological methods to identify microorganisms - from past to present. Microorganisms.

[17] Dunlap CA, Kwon SW, Rooney AP, Kim SJ (2015). *Bacillus paralicheniformis* sp. nov., isolated from fermented soybean paste. Int. J. Syst. Evol. Microbiol..

[18] Dunlap CA, Kim SJ, Kwon SW, Rooney AP (2016). *Bacillus velezensis* is not a later heterotypic synonym of *Bacillus amyloliquefaciens*; *Bacillus methylotrophicus*, *Bacillus amyloliquefaciens* subsp. *plantarum* and '*Bacillus oryzicola*' are later heterotypic synonyms of *Bacillus velezensis* based on phylogenomics. Int. J. Syst. Evol. Microbiol..

[19] Fan B, Blom J, Klenk HP, Borriss R (2017). *Bacillus amyloliquefaciens*, *Bacillus velezensis*, and *Bacillus siamensis* form an "Operational Group *B. amyloliquefaciens*" within the *B. subtilis* species complex. Front. Microbiol..

[20] Jang M, Jeong DW, Lee JH (2019). Identification of the predominant *Bacillus*, *Enterococcus*, and *Staphylococcus* species in meju, a spontaneously fermented soybean product. Microbiol. Biotechnol. Lett..

[21] Chang M, Chang HC (2007). Characteristics of bacterial-koji and doenjang (soybean paste) made by using *Bacillus subtilis* DJI. Korean J. Microbiol. Biotechnol..

[22] Cho MJ, Shim JM, Lee JY, Lee KW, Yao Z, Liu X (2016). Properties of meju fermented with multiple starters. Microbiol. Biotechnol. Lett..

[23] Hong Y, Jung HJ, Han SK, Kim HY (2016). Potentiality of *Bacillus amyloliquefaciens* KFCC11574P isolated from Korean traditional *doenjang* as a starter in the production of functional soya bean paste. Int. J. Food Sci. Technol..

[24] Ji WD, Yang SH, Choi MR, Kim JK (1995). Volatile components of Korean soybean paste produced by *Bacillus subtilis* PM3. J. Microbiol. Biotechnol..

[25] Lee KH, Choi HS, Hwang KA, Song J (2016). Quality changes in doenjang upon fermentation with two different *Bacillus subtilis* strains. J. East Asian Soc. Diet. Life.

[26] Yoo SK, Kang SM, Noh YS (2000). Quality properties on soy bean pastes made with microorganisms isolated from traditional soy bean paste. Korean J. Food Sci. Technol..

[27] Jeong DW, Lee B, Lee JH (2018). Complete genome sequence of *Bacillus licheniformis* 14ADL4 exhibiting resistance to clindamycin. Korean J. Microbiol..

[28] Jung JY, Chun BH, Moon JY, Yeo SH, Jeon CO (2016). Complete genome sequence of *Bacillus methylotrophicus* JJ-D34 isolated from *deonjang*, a Korean traditional fermented soybean paste. J. Biotechnol..

[29] Blom J, Albaum SP, Doppmeier D, Puhler A, Vorholter F-J, Zakrzewski M (2009). EDGAR: a software framework for the comparative analysis of prokaryotic genomes. BMC Bioinformatics.

[30] Aziz RK, Bartels D, Best AA, Dejongh M, Disz T, Edwards RA (2008). The RAST Server: rapid annotations using subsystems technology. BMC Genomics.

[31] Jeong DW, Heo S, Lee B, Lee H, Jeong K, Her JY (2017). Effects of the predominant bacteria from meju and doenjang on the production of volatile compounds during soybean fermentation. Int. J. Food Microbiol..

[32] Park KB, Oh SH (2006). Enhancement of γ-aminobutyric acid production in Chungkukjang by applying a *Bacillus subtilis* strain expressing glutamate decarboxylase from *Lactobacillus brevis*. Biotechnol. Lett..

[33] Le Vo TD, Kim TW, Hong SH (2012). Effects of glutamate decarboxylase and gamma-aminobutyric acid (GABA) transporter on the bioconversion of GABA in engineered *Escherichia coli*. Bioprocess Biosyst. Eng..

[34] Kurihara S, Oda S, Kumagai H, Suzuki H (2006). γ-Glutamyl-γ-aminobutyrate hydrolase in the putrescine utilization pathway of *Escherichia coli* K-12. FEMS Microbiol. Lett..

[35] Kurihara S, Kato K, Asada K, Kumagai H, Suzuki H (2010). A putrescine-inducible pathway comprising PuuE-YneI in which gammaaminobutyrate is degraded into succinate in *Escherichia coli* K-12. J. Bacteriol..

[36] Ammor MS, Mayo B (2007). Selection criteria for lactic acid bacteria to be used as functional starter cultures in dry sausage production: An update. Meat Sci..

[37] Tamang JP, Watanabe K, Holzapfel WH (2016). Review: diversity of microorganisms in global fermented foods and beverages. Front. Microbiol..

[38] Mujoo R, Trinh DT, Ng PKW (2003). Characterization of storage proteins in different soybean varieties and their relationship to tofu yield and texture. Food Chem..

[39] Qin L, Ding X (2007). Formation of taste and odor compounds during preparation of Douchiba, a Chinese traditional soy-fermented appetizer. J. Food Biochem..

[40] Sanjukta S, Rai AK (2016). Production of bioactive peptides during soybean fermentation and their potential health benefits. Trends Food Sci. Technol..

[41] Lioe HN, Selamat J, Yasuda M (2010). Soy sauce and its umami taste: a link from the past to current situation. J. Food Sci..

